# Validation of diverse and previously untraceable Sendai virus copyback viral genomes by direct RNA sequencing

**DOI:** 10.1128/jvi.00894-25

**Published:** 2025-07-31

**Authors:** Sarah E. Pye, Emna Achouri, Yanling Yang, Abdulafiz Musa, Carolina B. López

**Affiliations:** 1 Department of Molecular Microbiology, Washington University School of Medicine, St. Louis, Missouri, USA; 2 Center for Women’s Infectious Disease Research, Washington University School of Medicine, St. Louis, Missouri, USA; Emory University School of Medicine, Atlanta, Georgia, USA

**Keywords:** defective viral genomes, bioinformatics, LoCA, viral genomes, copyback viral genomes, direct RNA sequencing, paramyxovirus

## Abstract

Copyback viral genomes (cbVGs) are truncated viral genomes with complementary ends produced when the negative-sense RNA virus polymerase detaches from the replication template and resumes elongation from the nascent strand. Despite advances in methods to identify cbVGs based on the site of polymerase break and rejoin, PCR-based tools cannot provide full-length sequences of most cbVGs and/or can introduce errors and artifacts during cbVG amplification. These limitations have painted an incomplete picture of the diverse population of cbVGs generated during infection. To improve our ability to obtain native full-length sequences of cbVGs, we optimized direct RNA sequencing (DRS) as a fast and simple tool to sequence full-length cbVGs and harnessed a BLAST-based analysis approach to identify cbVGs from long-read sequencing data. We analyzed the DRS outputs of multiple Sendai virus (SeV) stocks to highlight both the utility and limitations of this tool. We found that to capture the dominant 546 nt cbVG produced by SeV strain Cantell, the length of complementarity between the virus trailer and the DRS oligonucleotide should optimally be increased to up to 32 nt. We also demonstrate comparable quality of cbVG sequences by DRS from as little RNA as 17.6 ng from the media fraction or 50 ng from the cellular fraction of cells infected with SeV, in contrast to the recommended 1,000 ng. Importantly, we validated different cbVG species from two recombinant SeV stocks, including cbVGs whose break positions occurred at or near position one in the reference genome.

**IMPORTANCE:**

Most viruses of the order Mononegavirales have been demonstrated to naturally generate copyback viral genomes. These genomes are critical determinants of infection outcomes; they interfere with standard virus replication by competing for viral resources, activate antiviral responses, and inhibit protein translation. Despite their critical roles in infection, current tools to study copyback viral genomes rely either on preexisting knowledge of the sequence of a target RNA or require reverse transcription and amplification of the target RNA, biasing toward short copyback genomes and introducing relatively high rates of errors. Here, we detail the optimization of direct RNA sequencing to validate native full-length copyback viral genomes, including species that have not been validated previously.

## INTRODUCTION

**Fig 1:**
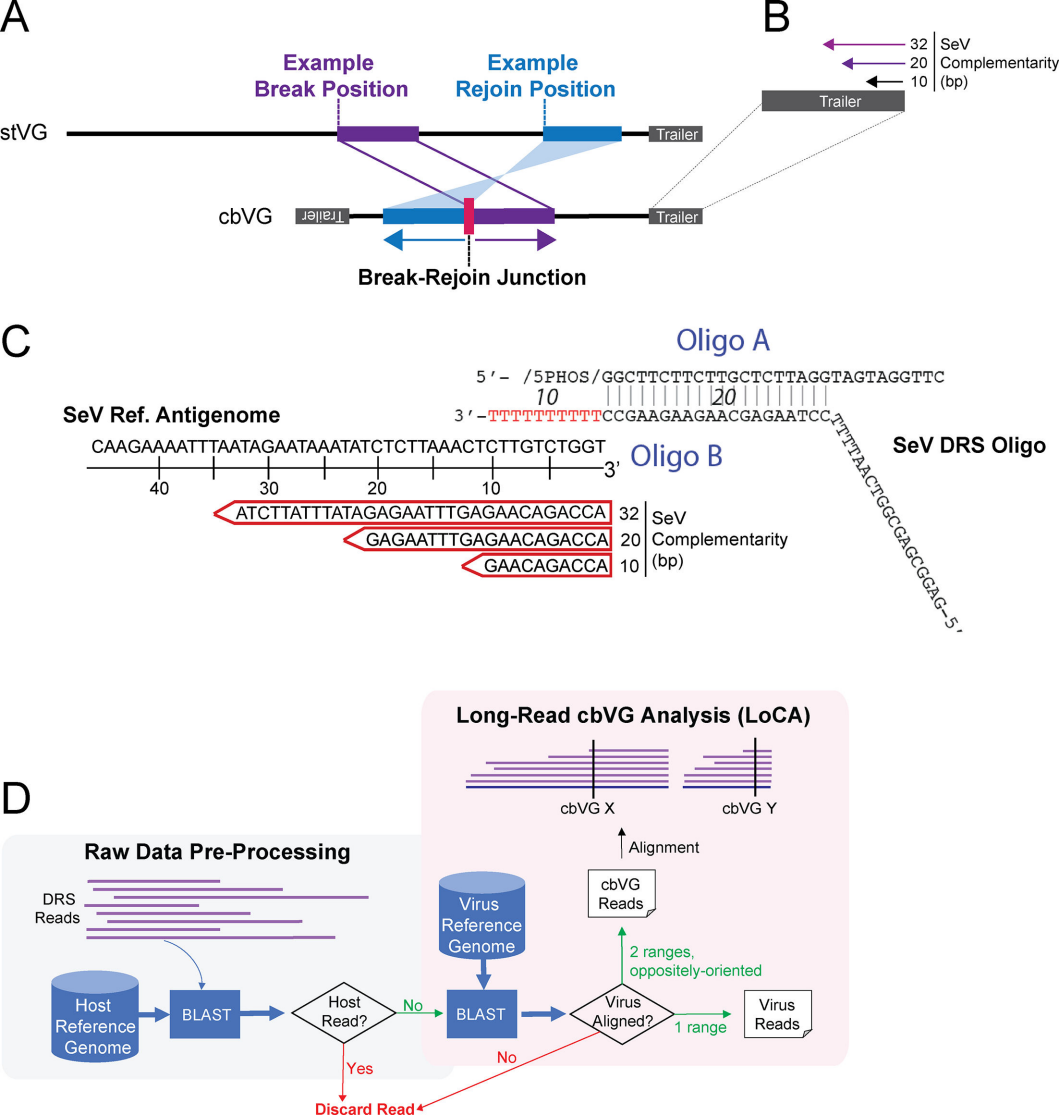
Long-read cbVG analysis (LoCA) script (https://github.com/lopezlab-washu/LOCA.git) identifies cbVGs from long-read direct RNA sequencing (DRS) data. (**A**) cbVGs are generated when the viral polymerase detaches from the replication template at a “break position” and resumes elongation downstream at a “rejoin position,” copying back a sequence complementary to the 5′ end of the nascent genome. Adapted from reference 4. (**B**) Sequence-specific adapter oligonucleotides of varying lengths were designed and tested against the Sendai virus (SeV) trailer for DRS. Oligo design in trailer permits cbVG capture independent of break-rejoin junction. (**C**) Specific sequences of the final DRS adapter oligonucleotide, where the 10 dT nucleotides in Oligo B were replaced by the corresponding SeV binding sites indicated by red arrows. (**D**) DRS analysis for cbVGs begins with the removal of host-aligned reads from the sequencing data. Next, non-host reads are aligned by BLASTn to the virus reference genome, from which reads aligned in one range are reported as virus reads and reads with two alignment ranges in opposite orientations are reported as cbVG reads. cbVG reads with common break-rejoin junctions are grouped into a single cbVG species.

Negative-sense RNA viruses generate a diverse population of viral particles defined by their genomic content. Viral genomes include the standard viral genomes copied exactly from the positive-sense antigenomic intermediate and replication-competent viral variants that harbor non-lethal mutations (1). RNA viruses also produce non-standard viral genomes (nsVGs), also known as defective viral genomes (DVGs), whose replication requires the standard virus polymerase (2, 3). In negative-sense RNA viruses, the majority of nsVGs are copyback viral genomes (cbVGs) generated when the viral polymerase detaches from the replication template at a “break position” and resumes elongation downstream at a “rejoin position,” copying back a sequence complementary to the 5′ end of the nascent genome (Fig. 1A, adapted from reference 4) (1). cbVG generation appears to be restricted to, but conserved among negative-sense RNA viruses, with cbVGs identified within every virus of the order Mononegavirales (1, 5). To understand how RNA viruses establish infections, promote transmission to other hosts, and are maintained within the host population, it is important to consider all components of the virus community, including both standard and non-standard viral genomes.

During infection, cbVGs interfere with standard virus replication by competing for viral resources, activating antiviral responses, and inhibiting protein translation (6–12). cbVGs have been shown to arise naturally *in vivo* during infection with respiratory syncytial virus (RSV) (13) and measles virus (14) and with Sendai virus (SeV) in mice (13, 15, 16). cbVG generation and accumulation have been correlated with robust antiviral responses to several Mononegavirales (13, 17–19). Importantly, the timing of cbVG accumulation during infection has been associated with infection outcomes. Detection of cbVGs early after RSV infection in children was associated with low viral load and mild disease, where worsened clinical outcomes were seen in patients who generated cbVGs later in infection or when cbVGs were present for prolonged periods (20). With mounting evidence that nsVGs are naturally produced and amplified during infection, cbVGs are proposed as agents for therapeutic interventions (19, 21–23).

To investigate the impact of specific cbVGs in an infection, one must first know which cbVGs are present. Existing strategies typically employ short-read next-generation sequencing to predict cbVG species, complemented by reverse transcription-PCR (RT-PCR) to validate them. cbVG detection from short-read sequencing data is based on the identification of reads containing a junction of two oppositely oriented regions of the reference genome, called a break-rejoin junction (Fig. 1A). While short-read sequencing is highly sensitive and has dramatically increased our ability to detect cbVGs from diverse samples, the requisite fragmentation and short-read length provide limited information on full-length cbVG sequences. In addition, like all RT-PCR-based methods, short-read sequencing relies on cDNA synthesis, which can create artificial chimeric cDNA from template switching during RT and mis-priming in PCR (24–26). Thus, cbVG identification is limited by artifacts introduced both during the initial identification of a cbVG from short-read sequencing and during its validation by RT-PCR.

By contrast, Oxford Nanopore Technologies (ONT) direct RNA sequencing (DRS) eliminates biases from reverse transcription and/or amplification and does not require fragmentation of the RNA, allowing for sequencing of full-length native RNA molecules. DRS sequences native RNA molecules as they pass through protein nanopores embedded in an electrically charged membrane, with each nucleotide characteristically disrupting the ionic current to generate sequencing reads.

To optimize DRS for sequencing of full-length cbVGs, we utilized SeV because it is a well-studied murine paramyxovirus related to human parainfluenza viruses (11, 15, 27, 28). Furthermore, we employed the historically available SeV strain Cantell because it naturally produces and accumulates one specific well-characterized cbVG of 546 nt (cbVG 546), which confers strong immunostimulatory ability to the virus and shuts off protein translation (15, 29–32). We detail the optimization of DRS for sequencing full-length cbVGs and apply these findings to gain novel insights into the cbVG populations of two virus stocks with diverse cbVG species. While DRS was limited in its ability to quantify absolute cbVG numbers, the tool successfully validated full-length and partial-length cbVGs of variable lengths. These included, for the first time, cbVGs with break and rejoin positions at opposite ends of the reference genome, in accordance with our previous predictions both from virus passaged *in vitro* and from clinical samples (33). We show that DRS is a fast and accurate tool for validation and characterization of cbVGs from virus stocks and infected samples.

## RESULTS

### DRS optimization for cbVG detection

Direct RNA sequencing kits sold by ONT include an adapter oligonucleotide that contains 10 dT nt, designed to bind the terminal 3′ end of mRNA, within the poly A tail. To capture cbVGs originating from the trailer, we designed our adapter oligonucleotide to bind the terminal nucleotides in the trailer sequence of the SeV antigenome. This positive-sense trailer sequence is present in all cbVGs regardless of their specific break-rejoin junction, as well as in the standard full-length viral antigenome (Fig. 1A). However, we noted that adapter oligonucleotides designed to target the terminal 10 nt of the antigenome would have several potential off-target binding sites within the SeV genome due to similarity in sequences. To investigate whether the adapter oligonucleotide could be optimized by increasing the oligonucleotide length, we designed and tested DRS adapter oligonucleotides with 10, 20, and 32 nt of SeV complementarity (Fig. 1B and C). Initial optimization experiments were performed at the manufacturer’s recommendation of 1,000 ng input (total) RNA, collected from SeV-infected A549 cells 24 hours post infection (hpi) at a multiplicity of infection (MOI) 1.5 TCID_50_/cell. To analyze DRS data, we used features of our published and extensively validated Viral Opensource DVG Key Algorithm 2 (VODKA2) pipeline (4), first developed to identify cbVGs from short-read sequences. In brief, our Long-Read cbVG Analysis script (LoCA; https://github.com/lopezlab-washu/LOCA.git) uses the final steps from VODKA2 (BLAST validation, filtering, and reporting) to analyze viral reads after host reads are filtered from DRS data. LoCA sorts and reports reads that align in exactly one range as virus reads and reads aligned in two oppositely oriented ranges as cbVG reads (Fig. 1D).

**Fig 2:**
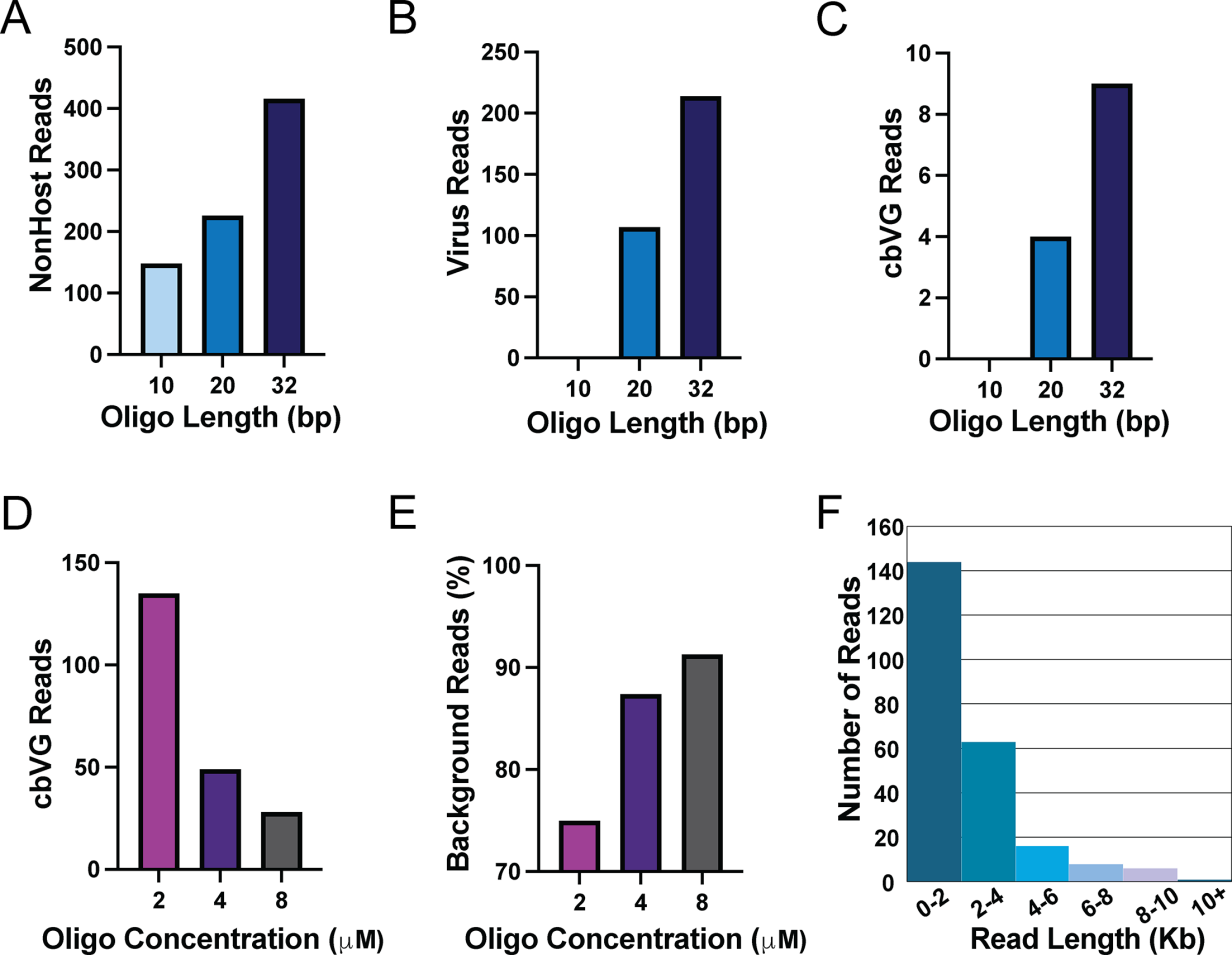
The length of sequence-specific oligonucleotide is critical for capture of cbVGs. DRS output from cellular RNA extracted from A549 cells 24 hours after infection by SeV Cantell at MOI 1.5. The library was prepared using sequence-specific adapter oligonucleotides with the indicated lengths of complementarity to the virus trailer and sequenced for 72 hours. Increasing the length of trailer-specific adapter oligonucleotide improved capture of (**A**) total (non-host) reads, (**B**) virus-aligned reads, and (**C**) cbVG reads. However, increasing adapter oligonucleotide concentration from 2 μM to 8 μM decreases capture of (**D**) cbVG reads and increases (**E**) background reads. (**F**) Histogram showing lengths of Sendai virus-aligned reads in an example DRS sample using 2 µM of a 32 nt trailer-specific adapter oligonucleotide. Read length bins of 2,000 nt.

First, we investigated the requisite length of virus complementarity within the adapter oligonucleotides for cbVG capture and indeed found that the 10 nt long adapter oligonucleotide was suboptimal to sequence cbVG 546. By contrast, longer adapter oligonucleotides of up to 32 nt increased each the total number of non-host (total) reads (Fig. 2A), virus reads (Fig. 2B), and cbVG reads (Fig. 2C). We next investigated whether increasing the concentration of the adapter oligonucleotide from the recommended 2 μM to 4 μM or 8 µM would increase the number of cbVGs but found that providing more adapter oligonucleotide decreased cbVG capture (Fig. 2D). In addition, excess adapter oligonucleotides during library preparation increased the frequency of background reads (non-host and non-virus aligned) (Fig. 2E). Furthermore, as expected when sequencing a stock that makes one short dominant cbVG, most reads in a representative sequencing experiment were shorter than 2 kB, but this strategy also captured some nearly full-length (15 kB) standard viral genome reads (Fig. 2F).

### DRS produces high-quality complete cbVG 546 sequences from little RNA

Next, we validated DRS in SeV infections of A549 cells over a time course, collecting both cellular fraction RNA (to capture active replication in the cells) and the more dilute media fraction RNA to reduce contamination by host RNA. DRS was successful in sequencing cbVGs from both sources, with cbVG levels consistently comparable between experiments beginning 24 hpi (Fig. S1A through C). The cbVG 546 sequences are high quality, with mismatches typically occurring as deletions within nucleotide repeats, also called homopolymers (Fig. S1D and E). This type of error is expected because cbVGs are highly structured and have shorter read lengths, resulting in more variable translocation speeds than standard viral genome (stVG). When sequencing long stretches of the same base by DRS, the signal does not change, so if the RNA translocation speed is not constant, the number of bases in homopolymeric regions can be misrepresented (34).

**Fig 3:**
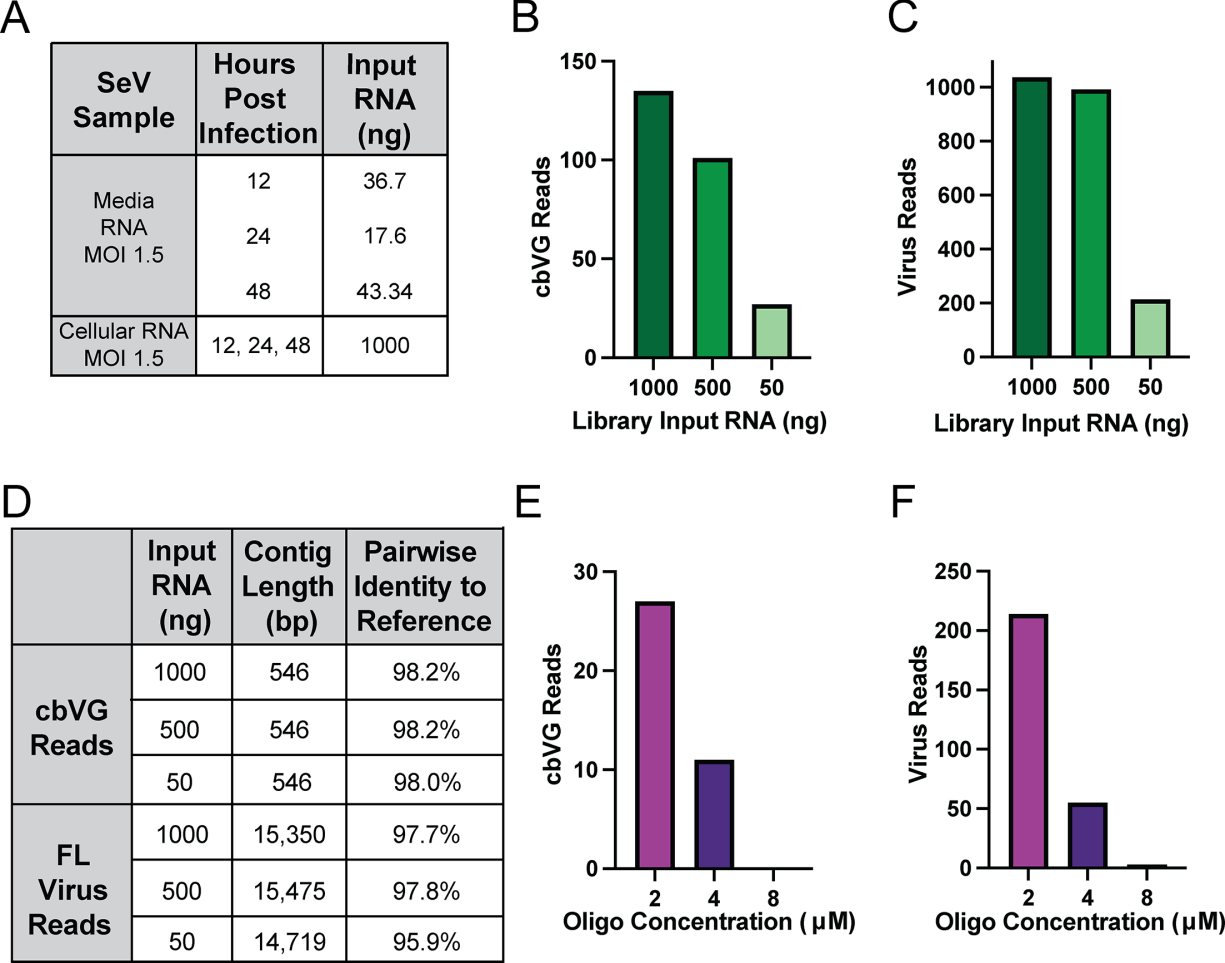
DRS accurately sequences full-length SeV copyback viral genomes from relatively little input RNA. (**A**) Full-length cbVG 546 was successfully sequenced at different time points from 1,000 ng cellular fraction RNA, or from as little as 17.6 ng media fraction RNA. (**B**) cbVG and (**C**) virus reads identified from cellular fraction RNA libraries at indicated input RNA quantities. (**D**) Percent identity and lengths of resulting cbVG and virus sequences for each condition of the cellular and media time course experiments in panels B and C. (**E**) cbVG and (**F**) virus reads from varying the concentration of trailer-specific adapter oligonucleotide from 2 μM to 8 μM while maintaining 50 ng library input RNA.

Of note, while all cellular RNA experiments sequenced a library made from 1,000 ng input RNA, DRS still produced high-quality complete cbVG 546 sequences from as little as 17.6 ng of RNA extracted from the cell culture medium (Fig. 3A). Since the media fraction is more dilute and has less host RNA than the cellular fraction, we wondered if cbVG sequencing will still be successful after decreasing the input RNA from the cellular fraction, where cbVGs are selected for among a greater population of potential contaminants. To test this, we decreased the cellular fraction input RNA to 500 ng and 50 ng and found reduced numbers of both cbVG and virus reads (Fig. 3B and C). However, pairwise identity was comparable between the cbVG sequences obtained from the 50 ng and 1,000 ng conditions (Fig. 3D). These results suggest that for most applications, just 50 ng of total RNA is sufficient for sequencing cbVGs. For assessments of pairwise identity, the DRS sequence was aligned to a full-length reference cbVG 546 sequence generated to contain every nucleotide from the end of the trailer until the break position, immediately followed by every nucleotide from the rejoin position back to the end of the trailer. As before, offering more adapter oligonucleotide during library preparation did not improve capture of cbVG or full-length virus (Fig. 3E and F).

### DRS validates diverse cbVGs predicted from short-read next-generation sequencing data

**Fig 4:**
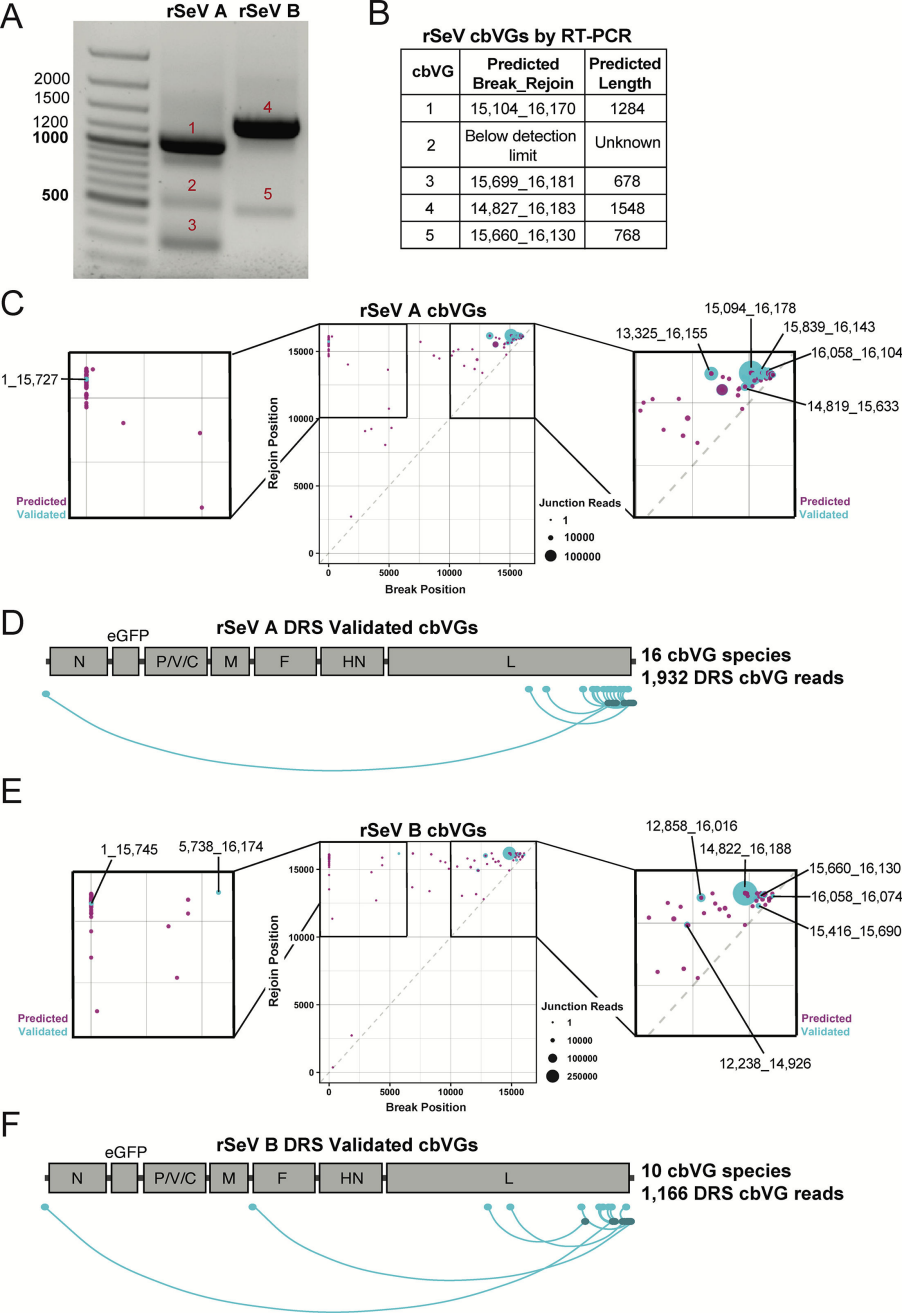
DRS validates cbVGs from rSeVA and rSeVB stocks. (**A**) Agarose gel (1%) highlights cbVG break-rejoin regions (partial-length sequences) amplified by cbVG-specific RT-PCR of rSeVA (left) and rSeVB (right) virus stocks. Bands from the gel were excised, purified, and sequenced. (**B**) Break-rejoin junctions and predicted full lengths of sequence-confirmed RT-PCR validated cbVGs. Numbering of break-rejoin junctions and predicted lengths of cbVGs assume synthesis of every nucleotide from the trailer until the break, and from the rejoin back to the end of the trailer of the SeV reference genome. (**C, E**) Total population of cbVGs predicted by VODKA2 with at least three reads from short-read next-generation sequencing of rSeVA and rSeVB stocks, respectively (magenta dots). DRS-validated cbVGs from each stock are highlighted in cyan and zoom views. (**D, F**) Virus gene annotations for break and rejoin positions of cbVGs validated by DRS at three or more reads from rSeVA and rSeVB stocks, respectively. cbVG schematic indicates break position in cyan, joined to the corresponding rejoin position in navy.

We next evaluated whether DRS can validate and provide complete cbVG sequences in a system with diverse cbVGs that includes longer species not previously validated. To do this, we employed two newly generated high-cbVG-producing stocks of rescued recombinant SeV, named rSeVA and rSeVB, which have cbVG profiles distinct from each other and from the cbVG 546-producing historical SeV Cantell stock described above. First, we used previously designed primers (15) to detect cbVGs in the rSeVA and rSeVB stocks by PCR, producing the bands in Fig. 4A. Whole plasmid sequencing after these bands were cloned into pGEM-T plasmids validated the break-rejoin junctions of four short cbVG species as listed in Fig. 4B. Of note, this method is often extremely low throughput, and these are partial sequences, limited by the location of the primers. Furthermore, the enzymatic nature of RT-PCR also biases shorter species, which can be limiting when validating very long cbVGs.

Thus, we initially predicted cbVGs from the rSeVA and rSeVB stocks by generating short sequencing reads that we analyzed by VODKA2 (4). By contrast to RT-PCR, analysis of short-read next-generation sequencing data by VODKA2 detected hundreds of thousands of cbVG break-rejoin junctions. Reads with similar break-rejoin junctions and lengths can be classified as cbVG species. We represented each cbVG species present in three or more short reads as a magenta dot whose size is correlated with its predicted frequency on a scatter plot. The axes of these scatter plots represent the position of each cbVG break (*x*-axis) and rejoin (*y*-axis) in the reference genome (Fig. 4C and E). These predicted cbVGs must undergo PCR-independent secondary validation, which has remained a challenge.

To validate cbVGs predicted by short-read next-generation sequencing, we sequenced the rSeVA stock twice and the rSeVB once by DRS, each time with 50 ng of RNA as input for the library. DRS-validated cbVG species (*N* ≥ 3 DRS reads) with shared break-rejoin positions ± 5 nt are highlighted in cyan in Fig. 4C and E for rSeVA and rSeVB, respectively. The rSeVA cbVGs encompassed the 15 most abundant predicted cbVGs, excluding #12 and including #23 (Fig. S2). From the rSeVB stock, DRS validated the nine most abundant predicted cbVGs, except for #8, and including cbVG-426, predicted at #133 in frequency from only three short reads (Fig. S3). In total, DRS sequenced 16 cbVG species from the rSeVA stock and 10 cbVG species from the rSeVB stock, whose annotated break and rejoin positions are summarized in Fig. 4D and F.

As expected from the short-read sequencing predictions, most of the validated cbVGs have break-rejoin junctions near the trailer end of the genome (Fig. 4D and F). In addition to these cbVGs, DRS enabled us to validate cbVG break-rejoin junctions in each sample characterized by distal break positions as far as position 1 in the reference genome and rejoin positions in or near the trailer (Fig. 4D and F). Although not every predicted cbVG was validated, all cbVGs sequenced by DRS were previously predicted from short-read next-generation sequencing. Complete lists of validated cbVGs and their properties are provided in Fig. S2 for rSeVA and in Fig. S3 for rSeVB.

Importantly, DRS cbVG reads are generally high quality, with an average quality score (*Q*-score) of 21 and corresponding basecalling accuracy of 99.2% from the pooled rSeVA and rSeVB DRS cbVG reads (Fig. S4A). When the minimum quality score threshold is lowered, cbVG read numbers increase overall, and many of the cbVG reads with lower quality scores are partial-length cbVGs (Fig. S4B). Full-length DRS-validated cbVGs ranged in length from 396 nt to 3,240 nt. These included all cbVG species validated by RT-PCR in Fig. 4A and B. Of note, all cbVGs sequenced at *N* ≥ 3 DRS reads followed the paramyxovirus “rule of 6” where the paramyxovirus genome size must be a precise multiple of six to be replicated efficiently (Fig. S2 and S3).

### DRS validates distal break-rejoin junctions

**Fig 5:**
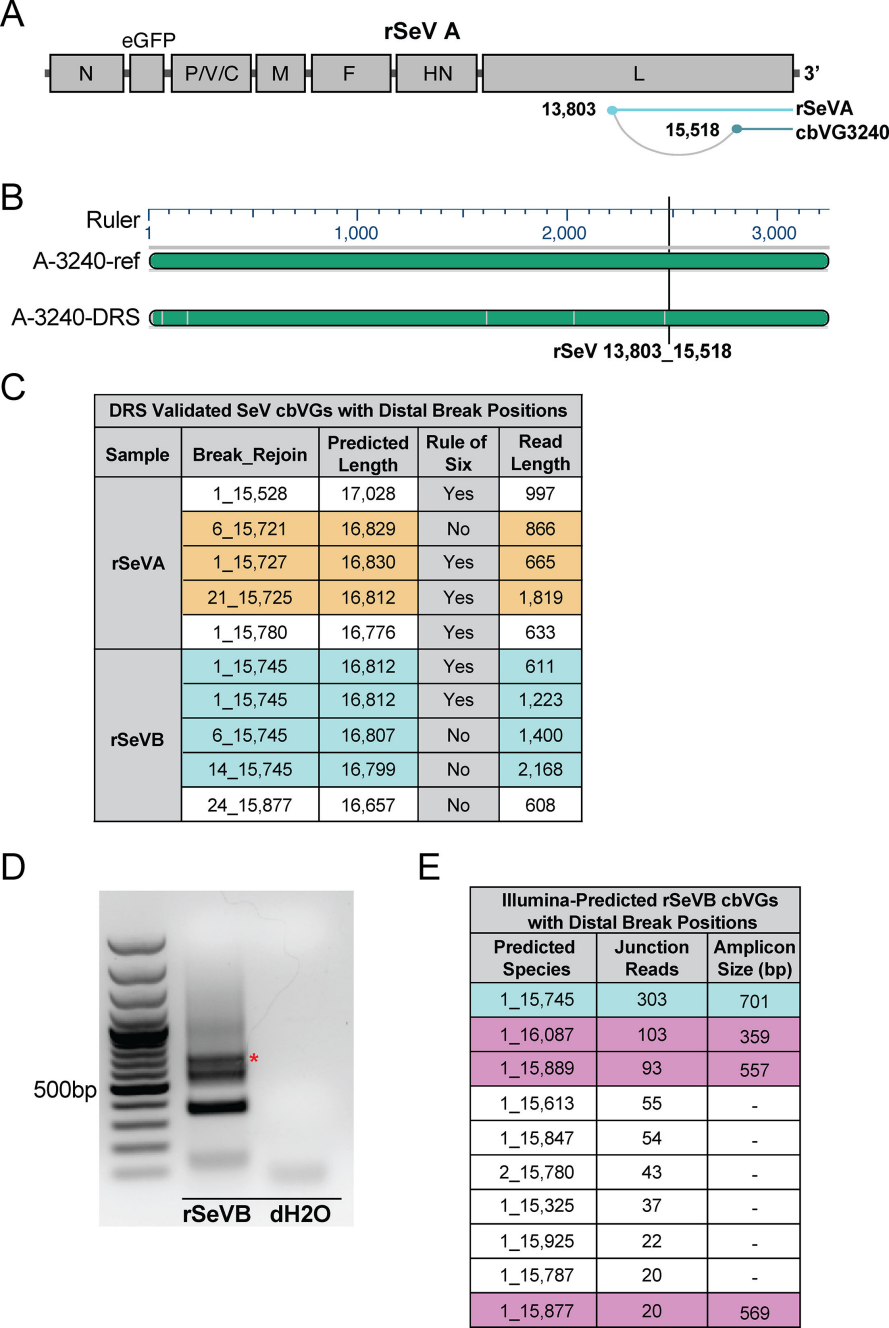
DRS validates the existence of long cbVGs by their break-rejoin junctions. (**A**) Schematic of the longest full-length cbVG validated by DRS, rSeVA-cbVG 3240 (named for its length). Sequence until cbVG break at rSeV position 13,803 is represented in cyan, with sequence from cbVG rejoin at rSeV position 15,518 until the end of the trailer is represented in navy. (**B**) Alignment overview of rSeVA-cbVG 3240 reference cbVG (top) to the DRS generated sequence (bottom). Gray vertical lines in DRS sequence indicate deletions from reference sequence (no substitutions or insertions were present). (**C**) DRS reads validate cbVGs with distal break positions at or near position 1 in the reference genome. Shown are the predicted complete length and actual read length of each cbVG with its validated break-rejoin junction, and whether it follows the paramyxovirus “rule of six” (genome length = 6*n* + 0). cbVG species are highlighted from each sample, grouped by shared rejoin position of ±6 nt. (**D**) Agarose gel shows amplicons resulting from RT-PCR to cross-validate cbVGs specifically with distal break positions. Red asterisk distinguishes 701 nt amplicon of DRS-validated cbVG 1_15,745. (**E**) Ten most predicted cbVGs with distal break positions. The table indicates cbVG species, junction reads, and amplicon size from panel D. Cyan highlight corresponds to the DRS-validated cbVG, while magenta highlights predicted cbVGs not validated in this DRS experiment.

Diagramed in Fig. 5A is the longest full-length cbVG sequenced, cbVG 3240 (from rSeVA, named for its full length in nucleotides). In general, cbVG sequence integrity by DRS is high; across all experiments, mismatches observed by DRS were typically deletions instead of substitutions. This holds true for cbVG 3240, where all 20 mismatches were deletions, often in regions of repeated nucleotides likely reflecting a technical limitation of DRS and not a biological feature of cbVGs (Fig. 5B). In the case of the longer validated cbVGs, the distance from the rejoin position until the end of the trailer, where the sequencing adapter oligonucleotide binds, is relatively much shorter than the distance before the break position. Thus, even partial length DRS cbVG reads directly validated highly diverse break-rejoin junctions, including cbVGs with predicted full lengths up to 16,830 nt.

Notably, from both rSeV stocks, DRS validated cbVGs characterized by a distal break at position 1 in the reference genome, and a rejoin in the final gene (L, polymerase gene) encoded by the virus genome. Some of these reads also follow the rule of 6, as highlighted in Fig. 5C. To orthogonally validate these cbVGs, we performed targeted RT-PCR to specifically capture rSeVB cbVGs with distal break positions, and Sanger sequenced the resulting bands. Indeed, we detected the DRS-validated cbVG with break_rejoin junction 1_15,745 (Fig. 5D, red asterisk). In the same reaction, other bands corresponded to other less prominent predicted cbVGs, also with distal break positions (Fig. 5E). In summary, we present two PCR-based methods, one with requisite fragmentation of RNA and one without, and one fragmentation-free PCR-free method, all cross-validating this cbVG. Previously, we have lacked adequate tools to validate whether these are true cbVGs or artifacts introduced during the sequencing library preparation, but DRS directly validates these potentially very long species, which have been predicted among clinical and complex infection samples (20, 33).

### DRS shortcomings

DRS would be maximally helpful to us as a quantitative tool to assess how many cbVGs are produced compared to standard viral genomes or to compare cbVG accumulation levels between species. However, even in the most optimal conditions tested, we found that a large percentage of the reads aligned to the host genome or were called as background reads. To dive deeper into this issue, we investigated which RNAs were represented in the reads from the 8 µM oligonucleotide condition, where nearly 80% of all sequenced reads align to the host genome (Fig. S5A). Among the 20% non-host reads, most reads aligned to the standard virus, with 7.4% cbVG reads and 29.4% background reads (non-host and non-virus aligned) (Fig. S5A). The host reads were enriched in the most prominent cellular RNAs in a typical A549 cell. Specifically, chromosome 17 is the most highly sequenced because of a polysomy of this chromosome in A549 cells (35), followed by ribosomal RNAs, long non-coding RNAs, and mitochondrial RNAs (Fig. S5B). Examination of the background reads in this sample showed that most cannot be classified as they are primarily composed of either G/C or A/U repeats (Fig. S5C). The background reads that could be manually classified included some very short reads aligning to common host RNAs (Fig. S5C). The high level of host reads sequenced from infections in A549 cells was not seen from infections in highly permissive monkey LLCMK2 cells (Fig. S5D).

**Fig 6:**
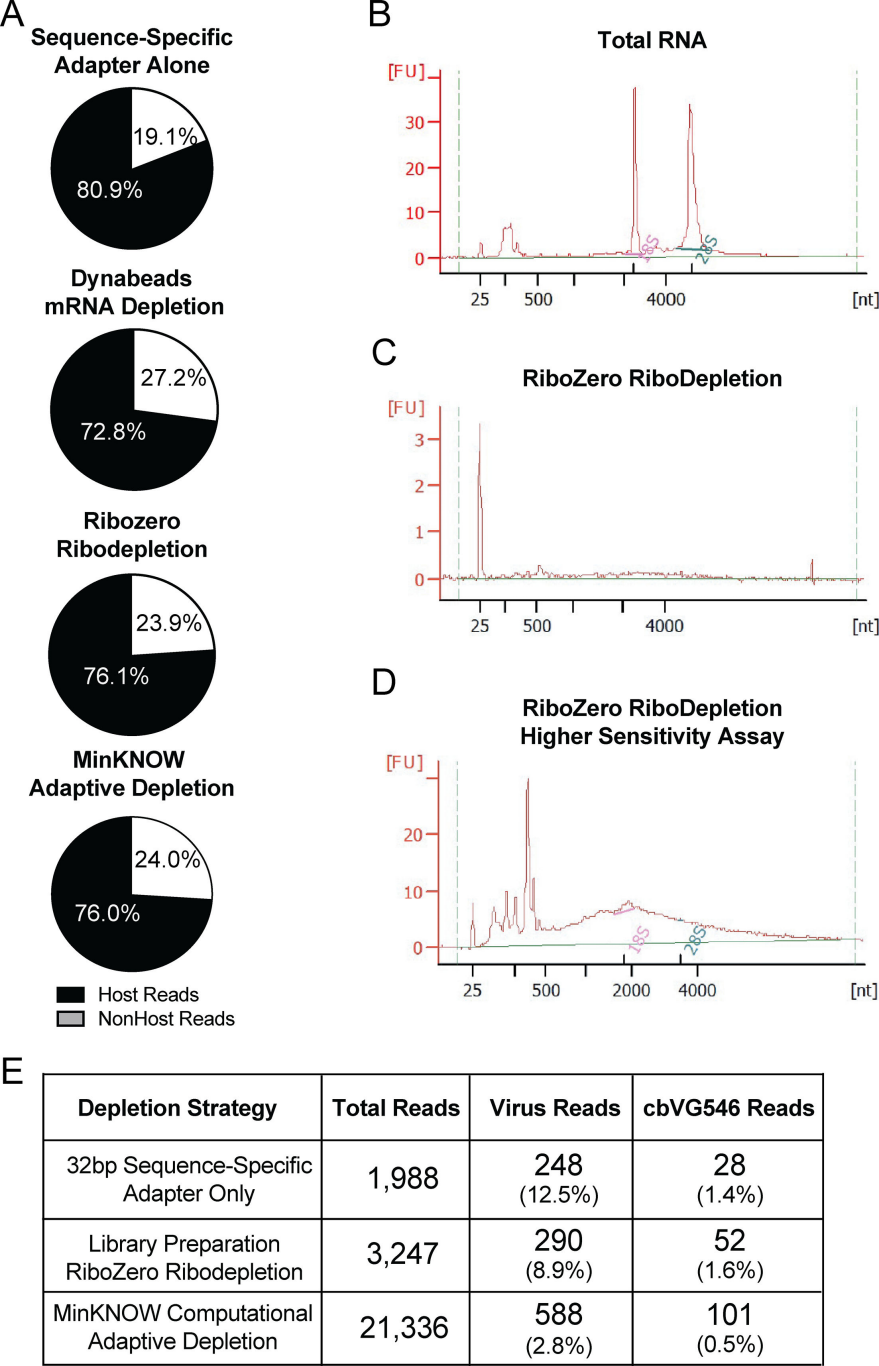
DRS in A549 cells is limited by nonspecific capture of host reads. (**A**) Percentages of host reads (black) and non-host reads (white) from sequencing DRS libraries prepared using the sequence-specific adapter alone, in addition to Dynabeads mRNA depletion or Ribozero ribodepletion, or from MinKNOW adaptive depletion computationally during data acquisition. (**B**) Bioanalyzer electropherogram traces from total RNA with 18S and 28S peaks labeled in pink and green, respectively. (**C**) Bioanalyzer electropherogram traces from Ribozero ribodepleted RNA. (**D**) Higher sensitivity bioanalyzer electropherogram traces from Ribozero ribodepleted RNA with 18S and 28S peaks labeled in pink and green, respectively. (**E**) Number of total DRS reads, virus-aligned reads, and cbVG reads from total RNA, Ribozero ribodepleted RNA, or by performing MinKNOW adaptive depletion computationally during data acquisition. Percentages of virus and cbVG 546 reads are shown below the read numbers in parentheses.

To reduce the proportion of host reads sequenced from A549-infected cells, we tested two methods for host RNA depletion before library preparation: Ribozero ribodepletion and Dynabeads mRNA depletion, as well as adaptive depletion through MinKNOW during sequencing for targeted depletion of the most common host reads sequenced previously. However, all these strategies similarly failed to significantly reduce the percentage of host reads in the sample (Fig. 6A). To verify successful ribodepletion, we confirmed by Bioanalyzer that the 18S and 28S peaks present in total RNA infected with SeV (Fig. 6B) are depleted after Ribozero ribodepletion (Fig. 6C), which can be confirmed using a higher sensitivity assay (Fig. 6D). Comparing Ribozero ribodepletion before library preparation to adaptive depletion during MinKNOW during data acquisition, we did not find proportionally higher levels of virus or cbVG reads, although we did sequence more reads overall compared to DRS without any host depletion, with increases in total numbers of both virus and cbVG reads (Fig. 6E).

## DISCUSSION

As cbVG generation is broadly conserved among the Mononegavirales, and cbVGs are highly influential in infections, it is critical that virologists have quick, simple, and reliable tools to identify cbVGs from virus stocks and infection samples. Long-read sequencing has emerged as a useful tool to sequence mRNA as well as viral RNAs. Deletion viral genomes from Flock House virus have been tracked using PCR-based long-read ONT sequencing (36). In addition, non-standard deletion viral genomes for coronaviruses and alphaviruses, and measles virus cbVGs from brain autopsies of patients who succumbed to subacute sclerosing panencephalitis (subsp.), have been identified by DRS (14, 37, 38). Of note, non-polyadenylated and highly structured cbVGs pose challenges for DRS, which was originally developed to sequence mRNAs. Here we presented a comprehensive optimization of DRS for cbVGs identification and described its application to simultaneously sequence multiple variable length cbVGs at the native RNA level without potential amplification-induced errors.

We found that to prepare successful libraries for DRS of cbVGs, users could increase the length of SeV complementarity in the sequence-specific adapter oligonucleotide up to 32 nt. We also found that preparing libraries with only 50 ng of input RNA is sufficient for most applications. Furthermore, we showed that depletion of host RNA, either by Ribozero ribodepletion or by MinKNOW adaptive depletion, increased the total number of sequenced reads and the number of both virus and cbVG reads, although it did not increase the percentage of virus or cbVG reads in the sample. In these cases, removing host reads from the sample that could otherwise occupy and clog nanopores enabled the sequencing of more RNA molecules in total, although this did not affect the proportion of host RNA, likely because the adapter oligonucleotide binds nonspecifically to prominent RNAs in the host cell. Together, this suggests that DRS is sufficient to sequence full-length cbVGs and stVG but is not yet quantitative.

There are several bioinformatic tools available to identify nsVGs from short reads, including DI-tector (39), DVG-Profiler (40), DG-Seq (41), ViReMa (36, 42, 43), DVGfinder (44), and VODKA2 (4). Among these tools, VODKA2 is optimized to identify cbVGs, with the lowest risk of detecting false positives (4). To generate a reliable tool for long-read sequencing data analysis, we isolated the final steps of BLAST validation, filtering, and reporting from VODKA2 to create the Long-Read cbVG Analysis script (LoCA; https://github.com/lopezlab-washu/LOCA.git). After host reads are filtered from long-read sequencing data, they are input into LoCA, which only requires a BLAST nucleotide database for the virus reference genome to classify virus and cbVG reads from DRS data.

Functionally, VODKA2 uses first Bowtie2 to align short reads to a catalog of hypothetical cbVG break-rejoin rejoins, then validates potential cbVG reads by BLAST (4). LoCA proceeds directly to the BLAST validation step, offering several advantages for long-read sequencing data that has fewer virus reads. First, there is reduced computational and labor demand because it is only necessary to generate and store a BLAST nucleotide database for the reference genome, not a catalog of hypothetical break and rejoin positions. This strategy is also unbiased and highly specific because it classifies cbVG reads based on alignment ranges instead of percent identity to a predicted reference. Finally, this also enables classification of partial or mutated reads; cbVGs can be reported independently of their predicted or actual length, and regardless of whether there are mutations or even truncations outside of the break-rejoin junction. In all the optimization and application experiments, cbVG reads reported by LoCA were true cbVGs; there was no evidence of artifactual recombination. One limitation of this study is that we analyzed LoCA reads with precisely two alignment ranges; we may have detected cbVGs with more complex architecture by investigating reads with more than two alignment ranges.

When we applied DRS to SeV stocks with more diverse populations of cbVGs, we validated 16 cbVGs from two sequencing experiments and 10 cbVGs from one sequencing experiment, each using only 50 ng RNA per library. In DRS, RNA molecules are translocated across the pore in a 3′ to 5′ direction but flipped during basecalling to generate 5′ to 3′ reads, so it is expected that the 5′ ends of some reads are missing as the most distal sequences supposed to pass through the pore can be lost due to RNA degradation or nanopore clogging. Full-length (>99%) DRS-validated cbVGs ranged in length from 396 to 3,240 nt, although partial length cbVGs with incomplete 5′ ends still validated diverse break-rejoin junctions. Among these partial length cbVGs, we validated species with distal break-rejoin junctions, specifically those with break positions at/near position 1 in the reference genome and rejoin positions in the trailer region.

Previously, we have reported cbVGs of a longer predicted size to predominate first after infection, prior to the diversification of cbVG species (33). Furthermore, these long cbVGs were present both *in vitro* in infections and in samples from RSV-infected patients (33). However, with few PCR-free tools available to validate them, it was important to discern whether they are artifacts introduced by template switching during RT (45). By cross-validating these predicted break-rejoin junctions using traditional RT-PCR and fragmentation-free amplification-free DRS, we provide the first compelling evidence of the verity of these cbVG-like genomic products. Functionally, we hypothesize that these very long cbVGs may be an intermediate species in cbVG diversification during infection (33).

One limitation of DRS is that cbVGs with a very short loop region, but very long complementary ends, would be more difficult to sequence, both because of their length and their likelihood to form secondary structure. As DRS technology continues to improve, we hope to validate and investigate this type of predicted cbVG species. Furthermore, the frequency at which a break-rejoin junction was sequenced by DRS did not always correspond with the frequency predicted by short-read next-generation sequencing, but we cannot conclude from our data whether this is representative of the actual amount of RNA present in the sample because we did not investigate the relative efficiencies of adapter oligonucleotide binding to different RNAs. Similarly, we cannot distinguish whether predicted cbVGs that were not validated by DRS were PCR-generated artifacts or simply below the limit of detection by DRS.

DRS addresses the need for an affordable and sensitive tool to validate cbVG sequences. This tool is not yet quantitative, but we have seen vast improvements in the DRS kits since their initial release, which make us confident that cbVG quantitation by DRS will eventually be possible. Our sequencing outputs for cbVGs continued to increase as new kit improvements were released; to sequence cbVGs by early versions of the DRS kit, we initially had to maximize RNA concentration and supplement additional adapter oligonucleotide in the library preparation. While we sequence most of the dominant predicted cbVG species, DRS does not always capture the lower frequency cbVGs. It is also apparent that cbVG secondary structure may affect DRS outcomes; beyond physically blocking the nanopores to decrease flow cell health during DRS, cbVG translocation speeds can be variable. Longer cbVG reads that have more nucleotides available to average this translocation speed tend to be higher quality reads.

Sequence validation of cbVGs is a critical first step to study the presence of or functional impacts of molecular motifs in cbVGs. Following the recommendations herein, virologists will now be able to use DRS to sequence full-length cbVGs in their samples and use LoCA to detect and extract those reads for further study. DRS is a critical supplement to existing tools in this field, both for cbVG specialists to uncover hidden sequence features of cbVGs and for non-specialists to discover dominant cbVGs with prospective significance in their systems.

## MATERIALS AND METHODS

### Cell lines and virus infection

A549 cells were cultured and infected with SeV as previously described in reference 46.

### rSeVA and rSeVB virus rescue and cbVG high stock development

Two independent rSeVC^eGFP^ viruses, named rSeVA and rSeVB, were rescued as previously described (46) and titered as described in https://doi.org/10.17504/protocols.io.ewov1d73yvr2/v1. cbVG high virus stocks were developed from the rescued viruses as described in https://doi.org/10.17504/protocols.io.j8nlk9xqwv5r/v1.

### RNA extraction

Total RNA was extracted from either the cellular or media fraction of infected cells as described in https://doi.org/10.17504/protocols.io.x54v922pml3e/v1. Cellular fractions were extracted in 1 mL TRIzol reagent while media fractions (1 mL) and virus stocks (500 µL) were extracted in TRIzol-LS reagent.

### cbVG RT-PCR and PCR band sequencing

SeV cbVG RT-PCR was done as described in https://doi.org/10.17504/protocols.io.5qpvok3j7l4o/v1. cbVG bands were cut and inserted into the pGEM-T vector as described in https://doi.org/10.17504/protocols.io.yxmvme389g3p/v1. The plasmids containing cbVG bands were then sequenced through Plasmidsaurus (Fig. 4) or by Azenta Genewiz Sanger sequencing (Fig. 5). RT-PCR to detect cbVGs with distal break positions was performed by the same protocol, but with the following primers:

RT primer: 5′-ACCAGACAAGAGTTTAAGAGATATGTATTC

PCR 1: 5′-GCTCCTCCTAGAGCTAAATGTATCG

PCR 2: 5′-ACCAGACAAGAGTTTAAGAGATATGTATTC

### RNA sequencing, RNA-seq data preprocessing, and VODKA2 cbVG detection

RNA sequencing library preparation was performed using Illumina TruSeq Stranded Total RNA kits and sequenced at 30 million reads per sample by RNA Seq NextSeq High output 2 × 150. As used to generate part of Fig. 4, data preprocessing and cbVG detection with VODKA2 were performed as described in reference 4.

### Direct RNA sequencing library preparation

DRS libraries were prepared using sequencing kit SQK-RNA004, following the manufacturer’s protocol and performing the optional SuperScript III reverse transcription step (version DSS_9197_v4_revA_20Sep2023-minion-2). The only modification we made is that unless otherwise specified, we designed our DRS adapter oligonucleotide to bind 32 nt of the SeV trailer, instead of only the recommended 10 nt (Table 1). The final DRS adapter oligonucleotide was annealed according to the manufacturer’s recommendation by mixing Oligo A and Oligo B in a 1:1 ratio in buffer (1.4 µM in 10 mM Tris-HCl, pH 7.5, 50 mM NaCl), heating to 95 °C for 2 minutes, and cooling slowly at 0.1 °C/second. Oligo A is an ONT-specified sequence, and the 10 dT of Oligo B sequence was replaced by the corresponding SeV binding sites in red (Table 1).

**TABLE 1:** SeV DRS adapter oligonucleotides

ONT DRS Oligo A: 5′-/5PHOS/GGCTTCTTCTTGCTCTTAGGTAGTAGGTTC	
Length of SeV complementary (nt)	DRS Oligo B sequences^a^
10	5′-GAGGCGAGCGGTCAATTTTCCTAAGAGCAAGAAGAAGCC(ACCAGACAAG)
20	5′-GAGGCGAGCGGTCAATTTTCCTAAGAGCAAGAAGAAGCC(ACCAGACAAGAGTTTAAGAG)
32	5′-GAGGCGAGCGGTCAATTTTCCTAAGAGCAAGAAGAAGCC(ACCAGACAAGAGTTTAAGAGATATTTATTCTA)

^a^ Sequences complementary to 3′ end of SeV trailer are underlined within parentheses.

SeV libraries were prepared using 1,000 ng total RNA unless otherwise indicated, while rSeV libraries were prepared using 50 ng total RNA (quantitation performed using a Qubit RNA HS assay kit on a Qubit fluorometer). All DRS experiments were sequenced for 72 hours on a MinION Mk1b using a new, previously unused FLO-MIN004RA, flow cell. Raw data sets were basecalled in MinKNOW at the high accuracy basecalling setting before passed reads were mapped to the human genome (GRCh38) using BLASTn. Non-host reads were extracted and used as input for the LoCA script, as described below. Following LoCA analysis, cbVG break-rejoin junctions represented in at least three DRS reads were classified as a single cbVG species.

### cbVG detection with LoCA

LoCA is freely available in GitHub (https://github.com/lopezlab-washu/LOCA.git). DRS reads were input into the LoCA script, which uses BLAST alignment to generate several files for each of the virus and cbVG reads: a text file with IDs of aligned reads, fastq and fasta files of classified reads, and a table report. The LoCA report summarizes the cbVG break-rejoin junctions, their predicted lengths, the size of the loop compared to the stem, the read sequence, and whether the cbVG follows the rule of 6 (can be modified or removed for viruses that are not constrained by this rule). Furthermore, cbVG reads are classified into common species with lengths and break-rejoin junctions separated by only ±5 nt. From the rSeVA stock, DRS validated 16 cbVG species from 1,932 total cbVG reads (97.7% of cbVG reads mapped into species). From the rSeVB stock, DRS validated 10 cbVG species from 1,166 total cbVG reads (99.1% of cbVG reads mapped into species).

### Alignment of cbVG reads

Alignment of cbVG reads for secondary analysis was performed in MegAlign software (DNAStar, Lasergene 17) using the MUSCLE algorithm. Comparisons of sequence identity were performed using the pairwise alignment function in MegAlign to align the consensus sequence from all cbVGs sharing a break-rejoin junction to a reference cbVG sharing the same break-rejoin position.

## Data Availability

Raw sequencing data of Sendai virus infections used herein are available in SRA under accession number PRJNA1232176.

## SUPPLEMENTAL MATERIAL

Supplemental material jvi.00894-25-s0001.pdf Figures S1 to S5.

ASM does not own the copyrights to Supplemental Material that may be linked to, or accessed through, an article. The authors have granted ASM a non-exclusive, world-wide license to publish the Supplemental Material files. Please contact the corresponding author directly for reuse.
