# Supplementary material for: Validation of diverse and previously untraceable Sendai virus copyback viral genomes by direct RNA sequencing
Source: J Virol. 2025 Jul 31;99(8):e00894-25. doi: 10.1128/jvi.00894-25 (PMC12363239; doi:10.1128/jvi.00894-25)

SUPPLEMENTAL MATERIALS

S1: Characterization of DRS cbVG 546 reads. Infections of A549 cells were performed using Sendai virus strain Cantell at MOI 1.5 for indicated time points. Libraries were prepared using sequence-specific adapter oligonucleotides with 32nt complementarity to the virus trailer and sequenced for 72 hours. **(A-C)** Virus and cbVG reads by sequencing libraries from either the cellular or media RNA fractions as indicated. **(D)** Pairwise alignment of the consensus sequence of cbVG 546 from cellular fraction RNA at 24hpi, obtained by aligning all SeV cbVG reads (top), to a reference cbVG 546 sequence (bottom). Mismatches in the DRS sequence are indicated as vertical grey lines against the green background. **(E)** Detailed pairwise alignment of DRS-generated cbVG 546 sequence (top) and reference cbVG546 sequence (bottom). Deletions and insertions are highlighted in grey while mismatches are highlighted in yellow. The break-rejoin junction of cbVG546 is indicated by the bold vertical red line.

S2: Overview report for all cbVGs validated by DRS from rSeVA. Chart shows the validated break-rejoin junction and predicted complete cbVG length, along with the number of reads by DRS, and whether it follows the paramyxovirus “rule of six” (genome length = 6n+0). Also reported are the percent of the complete cbVGs sequence validated by DRS, the percent identity of the sequenced portion of the cbVG, and a visual overview of the pairwise alignment between DRS consensus sequence and predicted reference sequence. Mismatched or un-sequenced bases are shown in grey.

S3: Overview report for all cbVGs validated by DRS from rSeVB. Chart shows the validated break-rejoin junction and predicted complete cbVG length, along with the number of reads by DRS, and whether it follows the paramyxovirus “rule of six” (genome length = 6n+0). Also reported are the percent of the complete cbVGs sequence validated by DRS, the percent identity of the sequenced portion of

the cbVG, and a visual overview of the pairwise alignment between DRS consensus sequence and predicted reference sequence. Mismatched or un-sequenced bases are shown in grey.

S4: cbVGs sequencing reads from DRS are high quality. (A) FastQC report showing quality per read of all DRS validated cbVG reads from both rSeVA and rSeVB stocks. Each sample basecalled at a minimum quality score threshold of 9 prior to fastQC analysis. **(B)** The quality score (Q-score) threshold can be modified by the user during basecalling and maintains or increases DRS read numbers for each break-rejoin junction species. Chart shows the NGS ranking corresponding to the relative frequency of detection of each break-rejoin junction by NGS, the predicted length, the number of DRS reads at the recommended quality score 9, and the number of DRS reads after reducing the quality score threshold to 7. The final column shows the percent increase in read counts by decreasing the quality score threshold to 7, colored as a heat map with the smallest increases in green and the largest increases in red.

S5: Characterization of DRS host and background reads. DRS output from cellular RNA extracted from A549 cells 24 hours after infection by SeV at MOI 1.5. Library was prepared using 8μM of 32bp trailer-specific oligo and sequenced for 72 hours. **(A)** DRS output consists of a mixture of host aligned and non-host aligned reads (percentages of total reads); the non-host reads can be further classified either as copyback viral genome (cbVG) reads, standard viral genome reads (virus), or non-host, non-virus aligned reads (background) (percentages of non-host reads). **(B)** BLASTn classification of host reads from the sample in panel A. **(C)** BLASTn classification of the Background (NonHost-NonViral) reads from panel A. **(D)** The percentage of host reads sequenced in a representative DRS experiment from Sendai virus infected LLCMK2 (black, left) and A549 (grey, right) cells.

Fig. S1

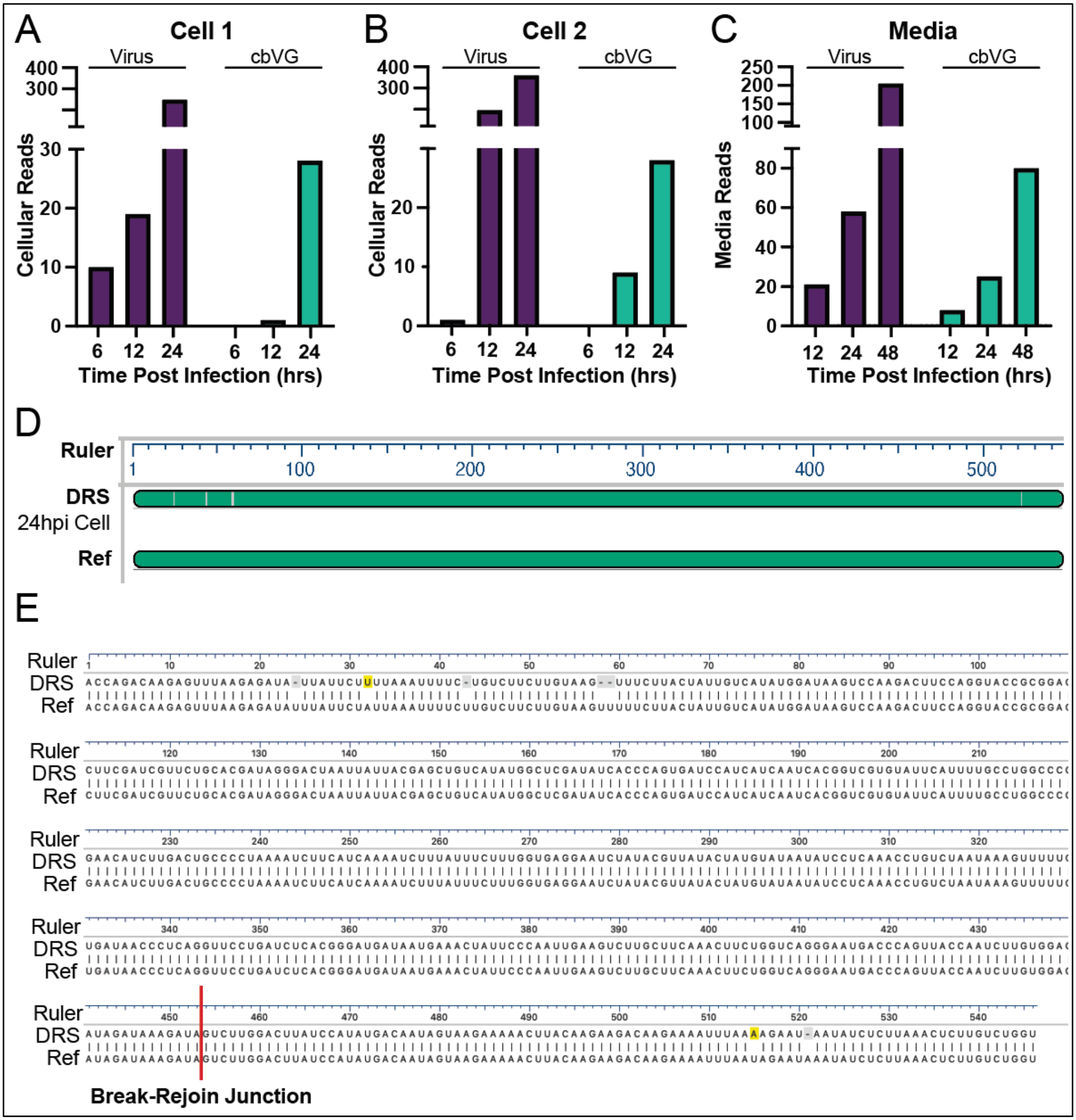

A
Cell 1
B
Cell 2
C
Media
Virus
cbVG
Cellular Reads
Media Reads
Time Post Infection (hrs)
D
Ruler
DRS
24hpi Cell
Ref
E
Ruler
DRS
Ref
Break-Rejoin Junction

Fig. S2

SeV Stock A cbVGs Sequenced by DRS

NGS Ranking	Break_Rejoin	Predicted Length	DRS Reads	Rule of Six	% Sequence Validated	% Identity	Alignment Overview of DRS and Reference Sequence
1	15,094_16,178	1284	434	Yes	100.0	99.5	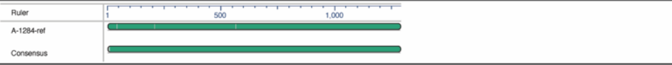
2	13,325_16,155	3078	182	Yes	100.0	99.6	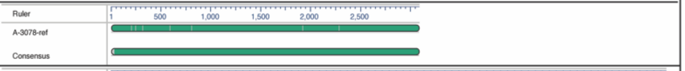
3	15,585_16,181	792	47	Yes	87.1	99.1	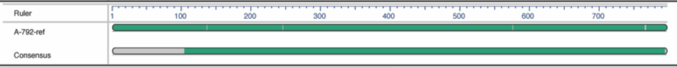
4	13,803_15,518	3240	227	Yes	100.0	99.5	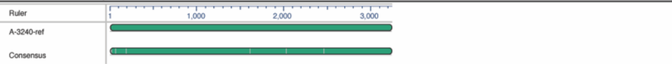
5	15,839_16,143	576	300	Yes	92.0	80.5	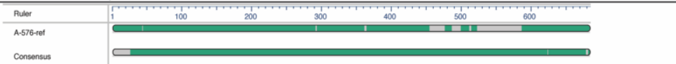
6	15,839_16,197	522	61	Yes	98.9	57.3	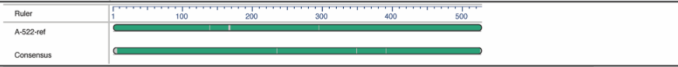
7	15,694_16,186	678	13	Yes	96.6	98.3	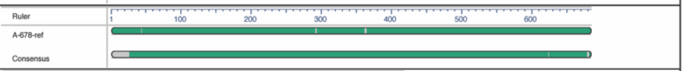
8	14,819_15,633	2106	21	Yes	100.0	98.9	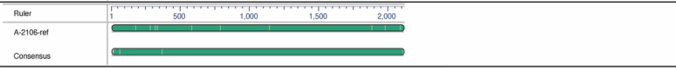
9	15,937_16,147	474	42	Yes	98.5	72.3	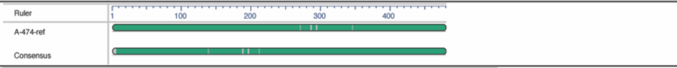
10	15,389_16,084	1086	14	Yes	100.0	97.9	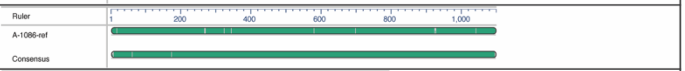
11	16,058_16,104	396	479	Yes	100.0	93.0	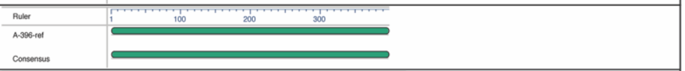
13	15,200_16,103	1254	17	Yes	100.0	97.7	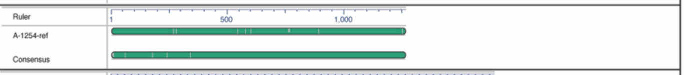
14	15,506_15,960	1092	3	Yes	33.0	96.0	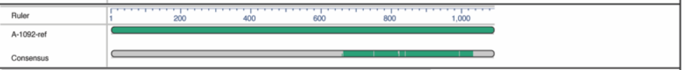
15	15,092_15,655	1806	3	Yes	74.0	72.6	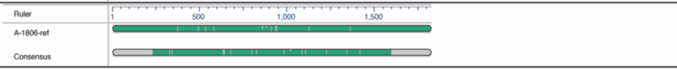
17	1_15,727	16,830	3	Yes	10.8	97.4	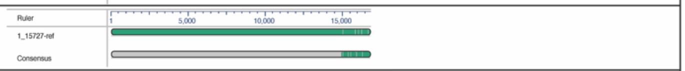
18	16,055_16,095	408	45	Yes	95.3	88.9	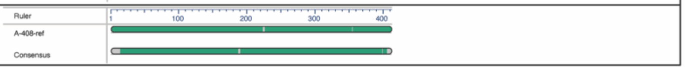

Fig. S3

SeV Stock B cbVGs Sequenced by DRS							
**NGS Ranking**	**Break_Rejoin**	**Predicted Length**	**DRS Reads**	**Rule of Six**	**% Sequence Validated**	**% Identity**	**Alignment Overview of DRS and Reference Sequence**
**1**	14,822_16,188	1548	1058	Yes	100.0	99.8	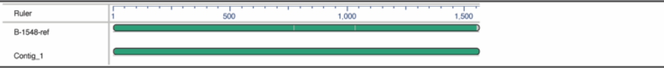
**2**	12,858_16,016	3684	25	Yes	95.4	99.0	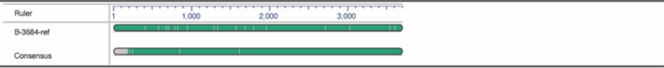
**3**	15,660_16,130	768	44	Yes	100.0	99.0	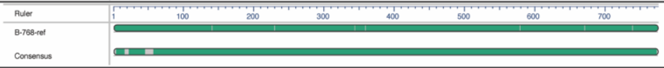
**4**	12,238_14,926	5394	3	Yes	41.5	95.5	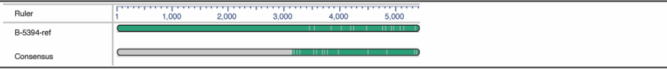
**5**	15,416_15,690	1452	10	Yes	90.1	86.6	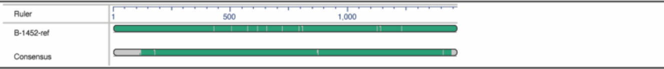
**6**	15,315_15,965	1278	5	Yes	86.5	96.9	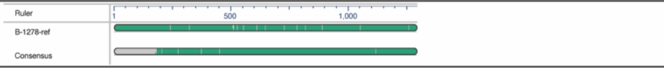
**7**	15,569_16,101	888	4	Yes	73.6	98.3	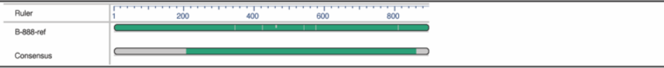
**9**	1_15,745	16,812	4	Yes	12.9	97.3	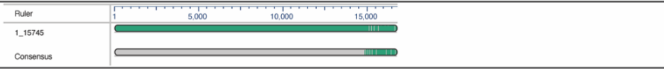
**12**	5,738_16,174	10,656	3	Yes	56.0	95.6	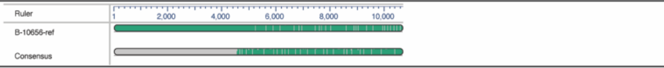
**133**	16,058_16,074	426	4	Yes	50.0	96.2	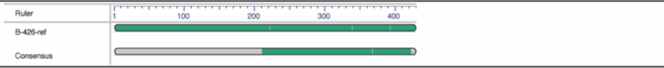

Fig. S4

A

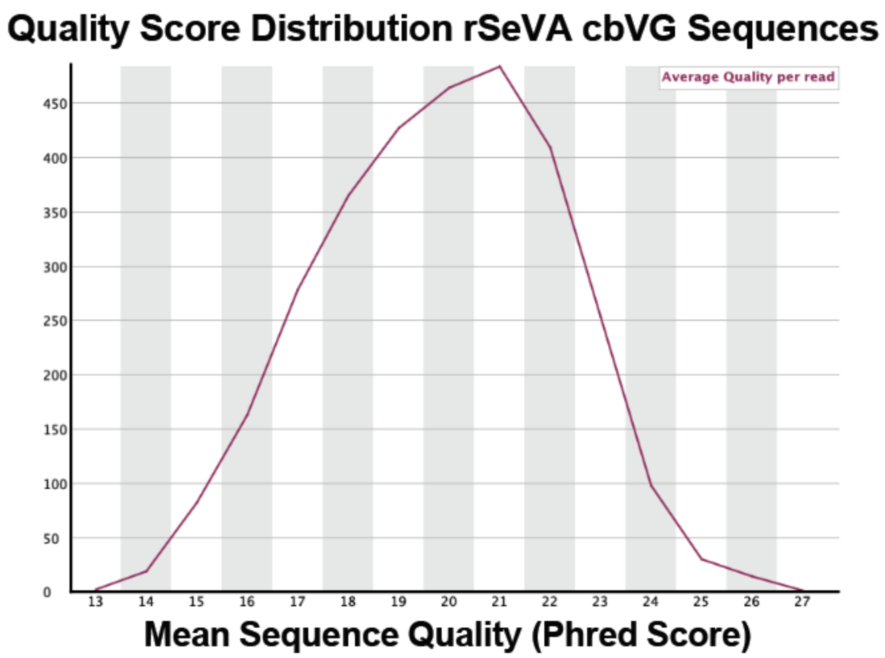

B

SeV Stock A - DRS Validated cbVGs by Quality Score					
**NGS Ranking**	**Break_Rejoin**	**Predicted Length**	**Q9 Reads**	**Q7 Reads**	**Percent (%) Increase**
**1**	15,094_16,178	1284	434	699	61.1
**2**	13,325_16,155	3078	182	351	92.9
**3**	15,585_16,181	792	47	71	51.1
**4**	13,803_15,518	3240	227	239	5.3
**5**	15,839_16,143	576	300	479	59.7
**6**	15,839_16,197	522	61	89	45.9
**7**	15,694_16,186	678	13	20	53.8
**8**	14,819_15,633	2106	21	22	4.8
**9**	15,937_16,147	474	42	81	92.9
**10**	15,389_16,084	1086	14	25	78.6
**11**	16,058_16,104	396	479	956	99.6
**13**	15,200_16,103	1254	17	23	35.3
**14**	15,506_15,960	1092	3	3	0.0
**15**	15,092_15,655	1806	3	6	100.0
**17**	1_15,727	16,830	3	3	0.0
**18**	16,055_16,095	408	45	98	117.8
**Total cbVG Reads**			1,932	3,278	69.7

Fig. S5

A

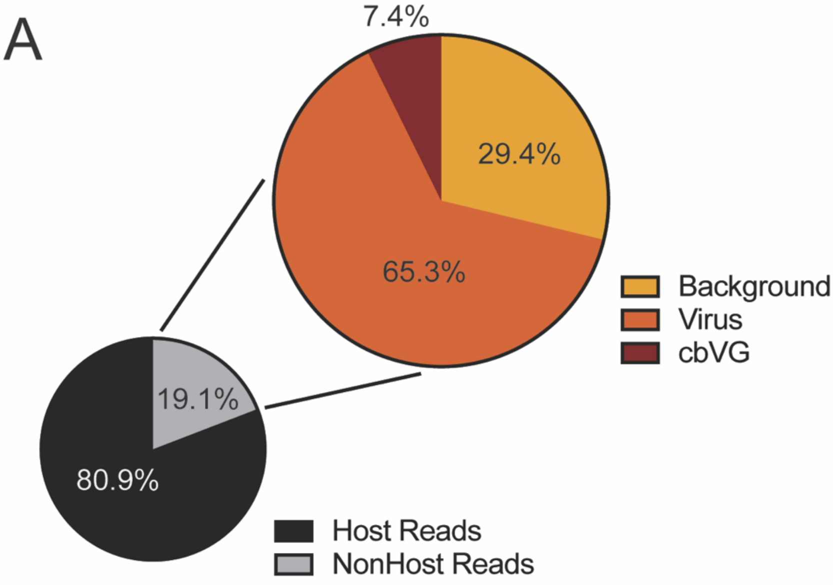

B

Host Reads

Reads	BLASTn Classification
965	Chromosome 17
240	28S Ribosomal RNA
172	18S rRNA
104	lncRNA
29	Mitochondrion
16	Other

C

Background Reads

Species	BLASTn Classification
1	No classification, GC-repeats
2	No classification, AU-repeats
3	Chromosome 17
4	lncRNA

D

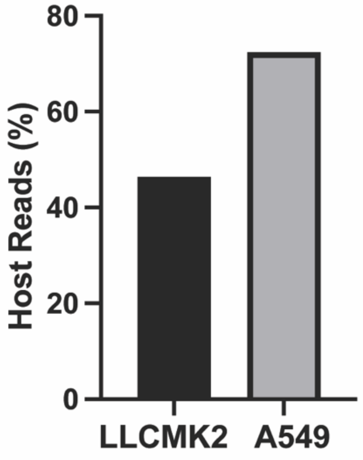

Supplement: Supplemental material — Figures S1 to S5. [file jvi.00894-25-s0001.pdf]
